# siMTA1-Loaded Exosomes Enhanced Chemotherapeutic Effect of Gemcitabine in Luminal-b Type Breast Cancer by Inhibition of EMT/HIF-α and Autophagy Pathways

**DOI:** 10.3389/fonc.2020.541262

**Published:** 2020-11-13

**Authors:** Pengping Li, Guodong Cao, Yuqing Huang, Wei Wu, Bo Chen, Zhenyu Wang, Maoming Xiong

**Affiliations:** ^1^Department of Thyroid and Breast Surgery, Department of General Surgery, First People’s Hospital of Xiaoshan District, Hangzhou, China; ^2^Department of Gastrointestinal Surgery, Department of General Surgery, The First Affiliated Hospital of Anhui Medical University, Hefei, China

**Keywords:** chemotherapy resistance, metastasis-associated protein1, epithelial mesenchymal transition, autophagy, exosomes

## Abstract

Advanced breast cancer holds a poor prognosis for chemotherapy and endocrine therapy resistance. Autophagy is one of the main causes of tumor drug-therapy failure, and increasing evidence shows that EMT also is responsible for that. Metastasis-associated protein1 (MTA1) is up regulated in lots of tumors, which leads to tumor progression and drug resistance. However, the role of MTA1 in chemotherapeutic resistance in luminal-b breast cancer is still unclear. In this paper, our research shows that higher expression of MTA1 accompanies with worse prognosis in luminal-b breast cancer. Knockdown of MTA1 enhances the sensitivity of MCF-7 to gemcitabine and weakens the metastasis ability of MCF-7 *in vitro* and *in vivo*. Further, we find that knockdown of MTA1 strengthens the gemcitabine-mediated tumor growth inhibition effect *in vivo*, through reversion of the EMT process and inhibition of the autophagy process. Furthermore, our research builds the siMTA1-loaded exosomes, which increases the gemcitabine-mediated tumor growth inhibition effect *in vivo*.

## Introduction

Breast cancer is the most common cancer in women, which causes the second most common tumor-related death events in females (1). Recurrence and metastasis are the key issues which lead to a 27% death rate in regression breast cancer (2). Chemotherapy is well-known as the main strategy to prolong the overall survival for advanced breast cancer, in which gemcitabine (GEM) and capecitabine are the alternative treatments for anthracycline and taxane chemotherapy failure. In addition, some small molecular inhibitors, such as CDK4/6 inhibitors, are also applied to prevent disease progression. However, the clinical outcome of those with advanced breast cancer is still unsatisfactory due to drug resistance induced by unclear factors, especially for the luminal-b type breast cancer and triple negative breast cancer (TNBC) which rely more on chemotherapy in the early stage of disease. So, a strategy to revert the chemotherapy resistance is pivotal in clinical treatment.

The mechanisms of chemotherapy resistance are complicated and are not yet fully understood. Autophagy is a necessity in normal body cells, which is mainly involved in cell metabolism and intercellular signaling process (3, 4). Frustratingly, autophagy is widely reported to be used to protect tumor cells from chemotherapeutic effects through down-regulation of oxidative stress and ER stress, up-regulation of mitochondrial function, and stress tolerance, among which the cellular pathways mainly contain PI3K/Akt/mTOR, MAPK/Erk/mTOR and p53/genotoxic stress (5–7). Some previous studies have applied inhibitors of autophagy, such as hydroxychloroquine (HCQ) and chloroquine (CQ) as a new strategy to deal with the chemotherapy resistance in clinical trials in glioblastoma, brain metastasis tumor, myeloma, advanced pancreatic cancer and lung cancer, which have gained considerable effects (8–11). Besides this, targeting pivotal proteins in the autophagy process, such as ATG5, ATG7 or beclin-1, is an another way to inhibit autophagy to sensitize the chemotherapy effect in cell or animal experiments (12).

The epithelial-mesenchymal transition (EMT) process is the first-recognized mechanism in tumor invasion and metastasis (13–15). Interestingly, recent studies imply that EMT is unnecessary and dispensable for tumor metastasis, but responsible for causing chemotherapy resistance, such as gemcitabine resistance in pancreatic cancer and cyclophosphamide resistance in breast cancer (16, 17). However, the mechanism of EMT-induced chemotherapy resistance is still elusive. Some previous studies point out that while undergoing the EMT process, tumor cells also initiate cancer stem cell (CSC)-associated signaling pathways, including Wnt, Hedgehog and Notch pathways (18). Some published research shows that EMT-associated transcriptional factors, including Slug and Zeb1, also participate in preventing the cellular apoptosis induced by EGFR-TKI in no-small cell lung cancer (19, 20). In addition, hypoxia is also involved in EMT-associated chemotherapy by HIF-α-induced up-regulation of MDR1 (21). However, single inhibition of EMT is not powerful enough to resolve the issue of chemotherapy resistance (21). So, the EMT-induced drug resistance still needs more investigation.

The metastasis-associated protein (MTA) family, containing MTA1, MTA1s, MTA1-ZG29p, MTA2, MTA3, and MTA3L, is famous for its member MTA1 which is first isolated from a metastatic breast cancer cell. MTA1 is an essential component of the nucleosome remodeling and histone deacetylation (NuRD) complex, which provides the opportunity for MTA1 to participate in the regulation of the MAPK/Erk pathway, Wnt pathway, DNA damage repairment, and ubiquitination pathway, which are closely involved in chemotherapy resistance, radiation resistance, tumor invasion, and regression in breast cancer, ovarian cancer, and colorectal cancer, (22, 23). Besides, the MTA1-NuRD complex can promote the EMT process by an up-regulation of the EMT-associated transcriptional factors, such as snail1, slug, Zeb1 and twist1 (23). In addition, a recent study implied that MTA1 overexpression also promoted the autophagy process, which lead tamoxifen resistance in luminal-b type breast cancer cell lines (24). Interestingly, a previous study also shows that MTA1 transferred by exosomes regulates the hypoxia process (25). So, blocking the MTA1 may be a useful strategy to sensitize the chemotherapy effects in advanced breast cancer. In addition, developed evidence suggests that exosomes are efficient carriers for drug delivery. So, our study hypothesizes that the exosomes can be a therapeutic carrier in MTA1-mediated drug sensitization.

In this study, we apply the TCGA database and clinical samples to analyze the relationship between MTA1 level and disease progression in luminal-b type breast cancer. We also explore the role of MTA1 in the gem-induced tumor toxicity effect *in vitro* and *in vivo* using small interfere RNA (siRNA) and the adenovirus stable knockdown system. In addition, we apply the siMTA1-loaded exosomes to sensitize the GEM-induced tumor cells death. Finally, we explore the mechanism of MTA1-induced chemotherapy resistance.

## Methods and Regents

The aim of this study is to explore the tumor growth inhibition effect of down regulation of MTA1 expression when it combines with gemcitabine in luminal-b type breast cancer and TNBC. All experiments are repeated at least three times in order to gain reliable data.

### Reagents

Gemcitabine was purchased from Ely Lilly (Bad Homburg, Germany) and was dissolved in sterile 0.9% sodium chloride. Bovine serum albumin (BSA) was purchased from Sigma-Aldrich.

### Breast Cancer Samples Preparation

This study was approved by The First Affiliated Hospital of Anhui Medical University Review Board and the ethics committees of Anhui Medical University. 188 paraffin-embedded tissue sections were collected from a tissue bank from January 2008 to January 2011. All patients with luminal-b breast cancer were confirmed by at least two pathologists.

### Cell Culture

Breast cancer cell lines (MDA-MA-231, MCF-7, 4T1) were gained from the cell bank of the Chinese Academy of Science in October 2017 with STR matching analysis. MDA-MA-231 was cultured in DMEM (Gibco, USA), MCF-7 and 4T1 were cultured in 1640 (Gibco, USA), and 293T was cultured in DMEM (Gibco, USA). All types of culture media were supplemented with 10% fetal calf serum and 100 units/mL penicillin and streptomycin.

### Cell Proliferation and Cytotoxicity Assays

The cell proliferation was quantified by standard curve (0.1, 0.2, 0.4, 0.8, 1.0, 1.5, 2.0, 3.0 x 10^5^ cells were detected at optical density (OD) *via* MTT 24h after being transplanted into 96-wells plates, and then the linear standard curve between log [cell quantity] and OD was fit), cell cytotoxicity assays were performed *via* MTT assay, and the detailed protocol described in our previous study (PMID:31935687).

### Clone Formation Assay

First, 0.2breast cancer cells (WT_MCF-7/MBA-MD-231, NC_MCF-7/MBA-MD-231 and siMTA1_MCF-7/MBA-MD-231) were seeded in 6-well plates for 2 weeks. Finally, cells were washed with PBS and fixed with 1 mL 4% formaldehyde solution. Then 1 mL crystal violet staining solution was added and washed with PBS 3 times after 30 minutes.

### Western Blot Analysis

Cells were harvested by cytology brush and lysed with RIPA lysis buffer (Sigma, USA) supplemented with phosphorylase and protease inhibitor mixture (Thermo, USA), quantified by the BCA assay. The standard detail experimental process of western blot was the same as our previous study (PMID:31935687). Western blot band was quantified through the Image-J software (NIH, USA). Antibodies against GAPDH, LAPM2 and p62 were purchased from Proteintech (1:1000, China), antibodies against Alex, c-myc, CD63, β-actin, BAX, BCL-2, LC3B, caspase3, caspase7, caspas8, caspas9, caspase12 and cleaved-PARP were purchased from Abcam (1:1000, China).

### Immunofluorescence Analysis

Briefly, 1 breast cancer cells were seeded in 24-well plates for 24h, followed with or without different treatments. Finally, cells were fixed by 4% paraformaldehyde, permeabilized by 0.5% Triton X-100, and blocked with 5% bovine serum albumin (BSA, Sigma) for 1 h at 37°C. Samples were incubated with primary antibodies (LC3B, LAPM2, or MTA1, 1:100) overnight at 4°C. Subsequently, they were washed by PBS, incubated with secondary antibodies for 1h at room temperature before being washed again. Finally, nuclei were stained with 15 μL DAPI (Sigma, USA) before being detected by fluorescence microscope.

### Immunohistochemistry Staining and Scoring Standard

Experimental procedure of paraffin embedding, tissue section, hematoxylineosin (HE) staining and immunohistochemistry for ki67 expression level were performed as previously described (PMID: 31935687). Furthermore, the working concentration of antibody against ki67 was 1:200 for proliferation index (Abcam). The protein expression level was assessed by Mean of Integrated Option Density (IOD) with Image-Pro^R^ Plus. Briefly, all of the Immunohistochemical sections were photographed for three yields in the same standard, and then Area of Interesting (AOI) was selected and IOD detected to gain Mean of IOD (IOD/AOI, MI).

### Migration Ability Assay

Migration ability assays contained transwell and wound healing assay. For transwell, 5.0 cells, with special treatments or not, were transplanted into transwell plates (24-well, 8.0μm, Corning Incorporated, Corning, NY, USA) with a 10% gradient of fetal calf serum for 48h. The detection procedure was the same as our previous study (PMID31935687). Quantification of passed cell area was performed by Image-Pro^R^ Plus. For wound healing assay, cells were seeded to at least 90% fusion in 6-well plates, and scratched by 200ul pipette tip, then washed with PBS to remove shed cells for extra 96h culture (PMID31935687). The scratch area was quantified with Image-Pro^R^ Plus.

### Tunnel Assay

The paraffin-embedded sections from animal-model-derived tumors were dewaxed in xylene for 5-10 minutes before switching to fresh xylene and dewaxing for another 5-10 minutes. Anhydrous ethanol was used for 5 minutes, 90% ethanol for 2 minutes, 70% ethanol for 2 minutes and distilled water for 2 minutes. 20 μg/ml of DNase-free proteinase K was added dropwise, and the reaction was affected at 20-37°C for 15-30 minutes. This was then washed 3 times with PBS or HBSS. Finally, maxing 2μl TdT enzyme + 48μl fluorescent labeling solution for each sample section. These sections were observed using an inverted fluorescent microscope.

### Intracellular ROS Production

Intracellular ROS production was detected by DCFH-DA probe. After being treated with drug, DMSO or 5μM GEM for 48h, 10 μL of DCFH-DA (2 mg/mL) was added to each group and this was future incubated for 30 min in the dark. After that, the cells were incubated in an incubator for 1 h and washed with PBS 3 times. Subsequently, 400 μL of DAPI (1 μg/mL) solution was added to the culture plates and maintained for 10 min. Finally, these cells were observed using inverted fluorescent microscope.

### Live&Death Cells Staining

The live&Dead cells staining was carried out using Calcein AM/PI staining. After being seeded in 24-well plate and cultured for 24h, breast cancer cells were treated with DMSO or 5μM GEM for another 48h. Then all cells were co-cultured with Calcein AM and PI and observed at 480 nm and 525 nm respectively.

### Harvesting of siMTA1-Loaded Exosomes

#### Part 1: Production and Collection of Exosomes

293T cells were cultured in 75 cm^2^ culture bottles to 80% of cell fusion in complete medium, then followed by 48h-culture in fetal-bovine-serum free medium. Then the supernatant was collected. Next, exosomes were concentrated and obtained: 1. The supernatant was centrifuged for 10 mins at 500G to remove the cell debris and large particle impurities; 2. The production from step-1 was centrifuged for 30 mins at 10000G to remove the large vesicles; 3. The production from step-2 was centrifuged for 12h at 100000G, 4°C, to obtain exosomes precipitation; 4. The exosomes precipitation was resuspended in RNase-free double distilled water (dd-water).

#### Part 2: Loading Into Exosomes of siMTA1

Firstly, the electroporation cuvette was pre-cooled for 45 minutes before electroporation. Then, 30ul exosomes (about 300ug/ml) and 10ul siRNA (20μM) were mixed in a microcentrifuge tube, and the volume was made up to 150 μL with citric acid buffer. Next, the mixture was transferred into an electroporation cup. The cuvette was then covered and placed in the cuvette holder of the electroporator. Finally, the electroporation program was selected and the start button pressed to start electroporation. (Note: “OK” appears on the display to indicate a successful pulse). When done, the cuvette was removed by turning the wheel 180° counterclockwise (PMID:30051423). Finally, the production was centrifuged for 12h at 100000G, 4°C, followed by gentle washing, using RNase-free double distilled water 1 time, which was finally followed by being resuspended in RNase-free double distilled water (dd-water).

### Uptake of siMTA1-FITC-Loaded Exosomes *In Vitro*

1 × 10^4^ breast cancer cells were seeded in 24-well plates for 24h, followed with treatment of 0.5ml siMTA1-FITC-loaded exosomes (20ug/ml) or 0.5ml exosomes (20ug/ml) for 12h, and then the uptake of exosomes was detected by fluorescence microscope.

### Experimental Protocols *In Vivo*

#### Part 1: Direct Delivery of GEM

The primary tumors were formatted by hypodermic injecting 1.0cells (NC_MCF-7 or siMTA1_MCF-7) in female athymic nude mice. After 4 weeks, as primary tumors grew into 1~2 cm (diameter), tumors were harvested and cut into 0.1 cm second generation tumors. Then the second-generation tumors were transplanted into female athymic nude mice (4 weeks), which were gained from the SLAC (Shanghai, China), and randomly divided into 2 groups (NC_MCF-7 or siMTA1_MCF-7 tumors were divided into 2 groups). Every 3 days, the weight of the mouse was tested. At day 16, 18 and 20, the first, second and third treatments of GEM or DMSO by intraperitoneal administration were given, testing tumor volume every 3 days from day 16. All tumors were harvested at day-26 to detect the tumor volume (MaAMiA^2^/2; MaA=Major axis, MiA=Minor axis) and tumor weight, followed by being processed into frozen sections for HE staining, tunnel assay and Ki67 staining.

#### Part 2—Delivery of GEM by Exosomes

The primary tumors were formatted by hypodermic injecting 1.0cells (WT_MCF-7) in female athymic nude mice. After 4 weeks, as primary tumors grew into 1~2 cm (diameter), tumors were harvested and cut into 0.1 cm second generation tumors. Then the second-generation tumors were transplanted into female athymic nude mice (4 weeks), which were gained from the SLAC (Shanghai, China), and were randomly divided into 3 groups. Every 3 days, the weight of mouse was tested. At day 16, 18 and 20, the first, second and third treatments of “A_vector + GEM”, A_siMTA1 or “A_siMTA1 + GEM” were given by intraperitoneal administration. All tumors were harvested at day 26 to detect the tumor volume (MaAMiA^2^/2; MaA=Major axis, MiA=Minor axis) and tumor weight, followed by being processed into frozen sections for HE staining and Ki67 staining.

### Statistics

All experimental data are presented as the means ± SD. Statistical Package for the Social Sciences version 21.0 (SPSS Inc., USA) was used for statistical analyses. ANOVA, paired t-test, Chi-square () test and nonparametric test (Mann Whitney U) were used for statistical analysis of different situations. Statistical significance was considered when p < 0.05 (*p < 0.05; **p < 0.01; ***p < 0.001; ns: p>0.05).All histograms and curves were constructed with GraphPad Prism 8 software (GraphPad Software, La Jolla, CA, USA).

## Results

### MTA1 Is a Risk Factor of Tumor Progression in Luminal-b Breast Cancer

**Figure 1:**
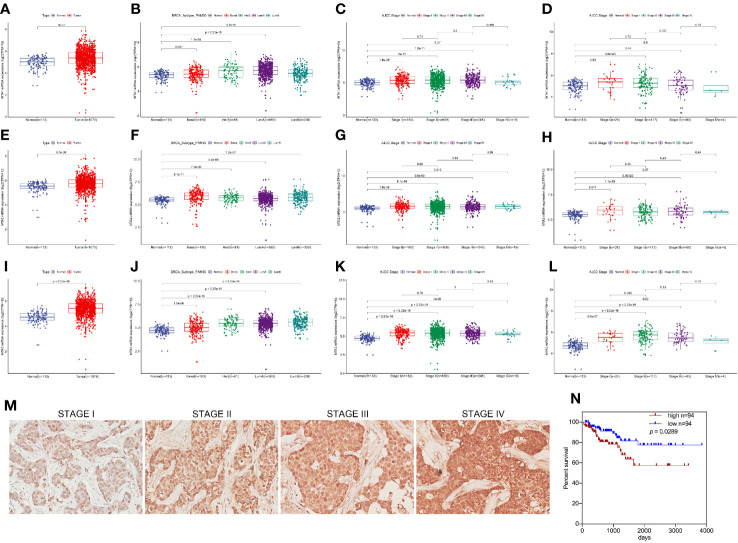
MTA1 is a prognosis factor of luminal-b breast cancer. (1) mRNA level of MTA1-3 in breast cancer tissues and adjacent normal tissues from TCGA data **(A, E, I)**. (2) mRNA level of MTA1-3 in breast cancer tissues (TNBC, Her-2 type, Luminal_A and luminal-b) and adjacent normal tissues from TCGA data **(B, F, J)**. (3) mRNA level of MTA1-3 in different clinical stages breast cancer tissues and adjacent normal tissues from TCGA data **(C, G, K)**. (4) mRNA level of MTA1-3 in different clinical stages luminal-b breast cancer tissues and adjacent normal tissues from TCGA data **(D, H, L)**. (5) IHC of MTA1 in luminal-b breast cancer tissue sections, which showed higher MTA1 expression with higher clinical stage (p<0.01) **(M, N)**. (6) IHC showed the overall survival in high and low expression of MTA1 breast cancer groups, which revealed higher expression of MTA1 accompanied with worse prognosis (p=0.0289) **(M, N)**.

First, in this study, we explore the relationship between the expression of the metastasis-associated protein (MTA) family and the prognosis of breast cancer. As the TCGA data shows, the mRNA level of the MTA family, containing MTA1, MTA2 and MTA3, is higher in breast tumor tissues than in normal tissues (p=1e-12 in MTA1, p=9.7e-9 in MTA2, p<2.22e-16 in MTA3; **Figures 1A, E, I**). We also found that the mRNA levels of MTA1-3 are up regulated in basal_like type, Her2 type, luminal_a type and luminal-b type breast cancer tissue, when compared with normal tissues (p<0.05, **Figures 1B, F, J**). In the further analysis, we found that there are no statistically significant differences about the mRNA levels of MTA1-3 in different clinical stages in all breast cancer samples or luminal-b breast cancer samples (**Figures 1C, D, G, H, K, L**). In the following, we verify the MTA1 level in 188 clinical samples, and the summary of the samples is shown in **Table 1**. However, in our own 188 clinical samples analysis, we found that the protein level of MTA1 is higher in clinical stage III~IV samples than in clinical stage I~II samples (52 of I~II and 42 of III~IV in high expression of MTA1 group, 66 of I~II and 26 of III~IV in high expression of MTA1 group, p=0.023, **Table 2**). That means the higher protein level of MTA1 accompanies the higher clinical stage, just as immunohistochemistry (IHC) shows in **Figure 1M**. The results of the Kaplan-Meier analysis of the 188 samples show that a higher protein level of MTA1 accompanies worse overall survival and increased mortality rates(p=0.029, **Figure 1N** and **Table 2**). In addition, we found that age is a factor affecting MTA1 level (p=0.015, **Table 2**).

**Table 1:** Summary of 188 luminal-b breast cancer samples.

Item		Cases	Percent (%)
**Gender**	**Male**	0	0.00
	**Female**	188	100.00
**Age**	**<46**	54	28.72
	**>45**	134	71.28
**Clinical Stage**	**I**	20	10.63
	**II**	109	57.98
	**III**	55	29.26
	**IV**	4	2.13

All 188 samples are from luminal-b breast cancer, from Jan. 2008 to Jan. 2011, gained from Anhui medical university affiliated NO.1 hospital.

**Table 2:** The expression of MTA1 in luminal-b breast cancer progression.

Item		Expression of MTA1		*P* Value
		High Expression	Low expression
**Age**	**<46**	19(20.2%)	35(37.2%)	0.015
	**>45**	75(79.8%)	59(62.8%)	
**Clinical Stage**	**I~II**	52(55.3%)	66(70.2%)	0.023
	**III~IV**	42(44.7%)	26(29.8%)	
**Survival**	**Mean value**	818.2	1088.6	0.029
	**Death Events**	21.3%	13.8%	

### Down-Regulation of MTA1 Sensitizes the Chemotherapy Effect of Gemcitabine in Luminal-b Breast Cancer Cells Rather Than in Triple Negative Breast Cancer Cells

**Figure 2:**
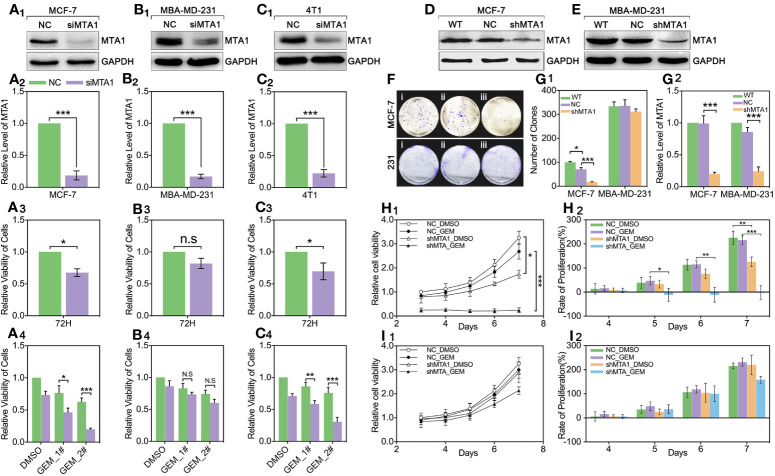
Down regulation of MTA1 sensitized gemcitabine-induced tumor growth regression in MCF-7 cell lines. (1) WB and statistics for small interfere RNA (siRNA) induced MTA1 knockdown in breast cancer cell lines: MCF-6, MBA-MD-231 and 4T1 **(A_1-2_–C_1-2_)**. (2) viability of cells, tested by MTT after 48h-treatment of siRNA and 72h culturing in 96-wells plates **(A_3_–C_3_)**. (3) cells are treated with siRNA for 48h followed by 48h-treatment of DMSO, 5μM gemcitabine or 10μM of gemcitabine **(A_4_–C_4_)**. (4) Stable knockdown of MTA1 in MCF-7 and MBA-MD-231 cells, verified by WB **(D, E, G_2_)**. (5) Cloning formation of WT_ MCF-7/MBA-MD-231(i), NC_ MCF-7/MBA-MD-231(ii) and shMTA1_MCF-7/MBA-MD-231(iii) cells **(F, G_1_)**. (6) NC_ MCF-7 and shMTA1_MCF-7 are treated with gemcitabine or DMSO for 48h, and then are cultured in fresh complete medium for 5 days, in which cells are tested for viability through MTT every day, and cell growth was inhibited as compared with NC **(H_1_, H_2_)**. (7) NC_MBA-MD-231 and siMTA_MBA-MD-231 cells are treated with gemcitabine or DMSO for 48h, and then are cultured in fresh complete medium for 5 days, which cells are tested for viability through MTT every day, and cell growth was inhibited as compared with NC **(I_1_, I_2_)**.

For exploring the role of MTA1 in regulating chemotherapy efficiency, our study applies small interfere RNA (siRNA), targeting MTA1 we named siMTA1, to block the expression level of MTA1. As **Figures 2A_1-2_–C_1-2_** show, 48h-treatment of siMTA1 efficiently down regulates MTA1 level in MCF-7, MBA-MD-231 and 4T1 (p<0.001). However, efficient down-regulation of MTA1 does not affect growth in mba-md-231 cells, but significantly inhibits growth in MCF-7 and 4T1 cell lines (p<0.05, **Figures 2A_3_–C_3_**). In the following experiments, we found that down-regulation of MTA1 enhances the gemcitabine (GEM, 48h treatment)-mediated growth inhibition more than 1-fold in 5μM or 10μM, when compared with the MTA1 normal expression group in MCF-7 and 4T1 cell lines, but not in the MBA-MD-231 cell line (p<0.05, **Figures 2A_4_–C_4_**). In order to verify those results, we established the MTA1-stable-knockdown cell lines in MCF-7 and MBA-MD-231. Just as the **Figures 2D, E, G_2_** show, MTA1 knockdown cell lines (shMTA1 cell lines) only hold about 20%~25% of the level of MTA1 when compared with NC cell lines (p<0.001).I In the 14-day clone formation test, we found that MTA1 knockdown significantly inhibited cloning cluster formation only in MCF-7 cell lines (p<0.001, **Figures 2F, G_1_**). Furthermore, we tested the long-term tumor growth inhibition effect of combining treatment of MTA1 knockdown and GEM. After a 48h-treatment of 5μM GEM in 96-well plates, the culture was refreshed with complete medium for another 5-day culture. As the data shows, MTA1 knockdown (shMTA1_DMSO) inhibited the cell growth more than 50%, when compared with the NC_DMSO group, only in the MCF-7 cell line (p<0.05, **Figures 2H_1_, I_1_**). MTA1 knockdown maintained the GEM-mediated cell growth inhibition effect after GEM withdrawal for 5 days, when compared with NC_DMSO, which only happened in the MCF-7 cell line (p<0.001, **Figures 2H_1_, I_1_**). In addition, MTA1 knockdown led to zero or even negative growth rate in MCF-7 cell lines (**Figure 2H_2_**), which did not occur in MBA-MD-231 cell lines (**Figure 2I_2_**).

### MTA1 Knockdown Raises the GEM-Mediated Ros-Reaction and Apoptosis in MCF-7 Cells

**Figure 3:**
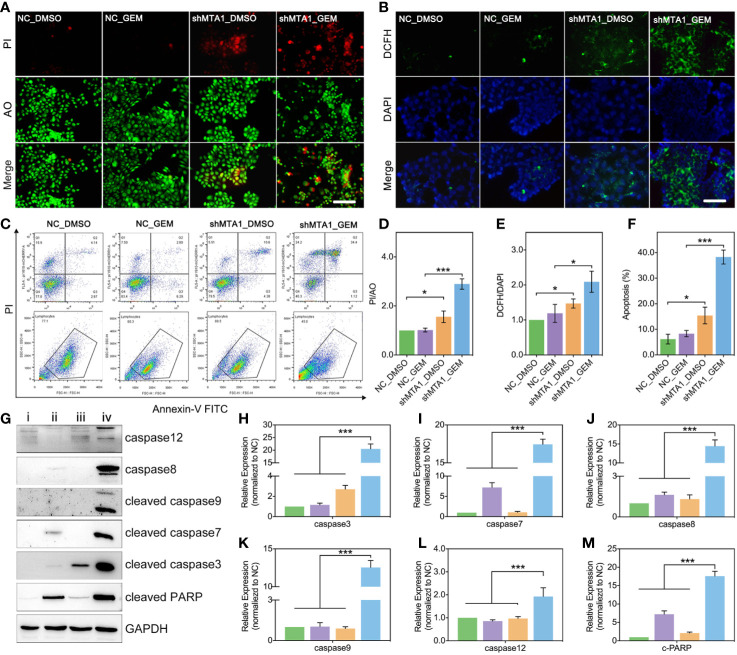
MTA1 knockdown with gemcitabine treatment up regulated the ros-reaction and apoptosis in MCF-7 cells. (1) breast cancer cells (NC_MCF-7 or shMTA1_MCF-7) are planted in 24-well plates for 24h, followed by 48h-treatment of 5μM gemcitabine or DMSO (4 groups: NC_DMSO, NC_GEM, shMTA1_DMSO and shMTA1_GEM), which are tested for live-death assay (live: green, death: red) **(A, D)**. (2) breast cancer cells (MCF-7 or shMTA1_MCF-7) are planted in 24-well plates for 24h, followed by 48h-treatment of 5μM gemcitabine or DMSO (4 groups: NC_DMSO, NC_GEM, shMTA1_DMSO and shMTA1_GEM), which are tested for ros-reaction through ros probes (ros: green, DAPI: blue) **(B, E)**. (3) breast cancer cells (MCF-7 or shMTA1_MCF-7) are planted in 6-well plates for 24h, followed by 48h-treatment of 5μM gemcitabine or DMSO (i: NC_DMSO, ii: NC_GEM, iii: shMTA1_DMSO, iv: shMTA1_ GEM), which are tested for apoptosis and WB assay **(C, F–M)**.

In this study, we found MTA1 knockdown enhanced GEM mediated cell death (AO/PI: death/live cell) more than 2-fold, compared with the NC_GEM group, through live&death assay (red: death cell, green: live cell) (p<0.001, **Figures 3A, D**). In addition, MTA1 knockdown enhanced GEM mediated ros-reaction (ros: green, DAPI: blue) more than 1.5-fold, compared with the NC_GEM group (p<0.05, **Figures 3B, E**). In order to quantify the cell apoptosis level, we used flow cytometry, which showed about 40% apoptosis level, which was only about 10% in the NC_GEM group (p<0.001, **Figures 3C, F**). Meanwhile, our study verified the above result *via* western blot (WB). As the **Figures 3G-M** show, combining treatment (“iv” group, MTA1 knockdown and GEM) obviously up-regulates the protein levels of caspase12, caspase8, cleaved-caspase3, cleaved-caspase7, cleaved-caspase9 and cleaved-PARP at least 1-fold(p<0.001).

### MTA1 Knockdown Enhances Gemcitabine-Induced Tumor Regression *In Vivo*

**Figure 4:**
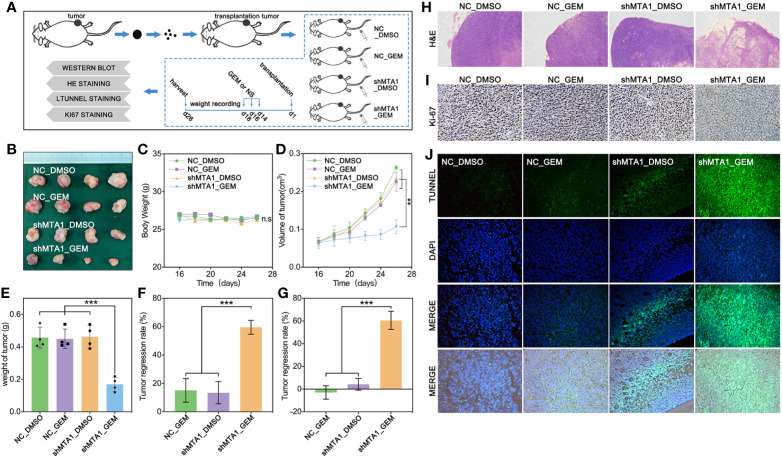
MTA1 knockdown enhanced gemcitabine mediated tumor growth restriction effect *in vivo*. (1) Experiments processed *in vivo* **(A)**. (2) Volume of subcutaneous transplantation tumor **(B)**. (3) Weight of mouse **(C)**. (4) Volume of tumors *in vivo* **(D)**. (5) Weight of tumors **(E)**. (6) Calculation of tumor growth inhibition by volume **(F)** and weight **(G)**, respectively. (7) H&E staining **(H)**, ki-67 staining **(I)** and tunnel assay, in which the green area means death **(J)**.

In order to test the function of MTA1 regarding regulating GEM resistance *in vivo*, our study establishes a subcutaneous xenograft modeling in female nude mice, and the process is showed in **Figure 4A**. Our experiments data show that the shMTA1_GEM group mouse held the smallest volume of tumor with indifferent weight of mouse when compared with the other three groups (NC-DMSO, NC_GEM and shMTA1_DMSO) (**Figures 4B, C**). Meanwhile, the tumor growth curve shows the slowest growth speed of MTA1_GEM as compared with the other three groups (p<0.01, **Figure 4D**). Other than that, tumor weight analysis shows that shMTA1_GEM held the slightest tumors (p<0.001, **Figure 4E**). We got the same results that combining treatment significantly increased the tumor growth inhibition rate, more than 2-fold, when compared with NC_GEM and shMTA1_DMSO, in weight and volume analysis (p<0.001, **Figures 4F, G**). In the following H&E staining, our data show that the shMTA1_GEM group held little tumor parenchymal cells staining, but more transparent area, which means an improved tumor cell death rate (**Figure 4H**), and the ki-67 staining held the same behavior (**Figure 4I**). For further verification, our study performed a tunnel assay, through which we got the same results that MTA1 knockdown endows a powerful chemotherapeutic sensitivity for GEM in luminal-b breast cancer cells. In other words, combining treatment increases the tumor growth inhibition (**Figure 4J**).

### siMTA1-Loaded Exosomes Combining With Gemcitabine Limits Tumor Growth *In Vivo*

**Figure 5:**
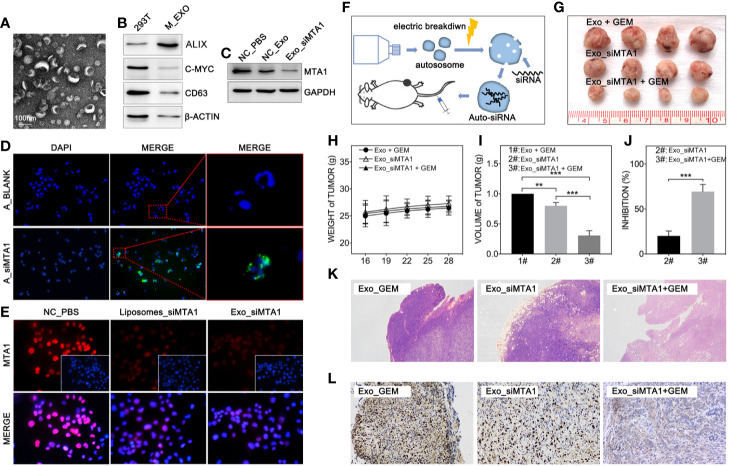
MTA1 loaded exosomes enhanced gemcitabine mediated tumor growth inhibition *in vivo*. (1) Electron microscopy of exosomes **(A)**. (2) WB assay tests markers of exosomes **(B)**. (3) WB assay for MTA1 knockdown efficiency by siMTA1-loaded exosomes (Exo_siMTA1) **(C)**. (4) Immunofluorescence (IF) assay for up taking of siMTA1-loaded exosomes by MCF-7 cells **(D)**. (5) IF assay tested MTA1 knockdown efficiency by siMTA1-loaded liposomes (LIP_siMTA1) and Exo_siMTA1 **(E)**. (6) Experiments process *in vivo* **(F)**. (7) Tumor volume (1#: Exo + GEM, 2#: Exo_siMTA1, 3#: Exo_siMTA1 + GEM) **(G, I)**. (8) Weight of mouse **(H)**. (9) **(J)** Tumor growth inhibition rate (2#: Exo_siMTA1, 3#: Exo_siMTA1 + GEM). (10) H&E staining **(K)** and ki-67 staining **(L)**.

Clinically, gene-targeted therapies have been carried out on HER-2 positive breast cancer, such as trastuzumab and pertuzumab, which have demonstrated strong tumor treatment capabilities. But for HER-2 negative breast cancer (luminal type or TNBC type), efficient chemotherapeutic regiments are still needed. Recently, small interfere RNA loaded drug systems have been used in preclinical experiments for tumor treatment, which show considerable therapeutic effect (26–28). Meanwhile, exosomes, an emerging chemotherapeutic drug delivery vehicle, have been applied in siRNA delivery (29). So, our study uses 293T-derived exosomes as the vehicle of siMTA1. The **Figures 5A, B** show the exosomes’ behavior. And as the **Figure 5D** shows, siMTA1-loaded exosomes (Exo_siMTA1) are successfully ingested by MCF-7 cells. In the WB assay, our data show the efficient of down-regulation of MTA1 by Exo_siMTA1 (**Figure 5C**). Furthermore, we found that Exo_siMTA1, when compared with liposomes, held comparable capability in MTA1 knockdown (**Figure 5E**). Next, we verified the Exo_siMTA1 *in vivo*. **Figure 5F** shows the process of animal experiments. And the **Figures 5G–J** show that the “Exo_siMTA1 + GEM” group significantly restricts the tumor growth, when compared with “Exo +GEM” group, which implies enhanced the chemotherapy sensitivity of MCF-7 to GEM by Exo_siMTA1 (p<0.001). In the H&E staining and ki-67 staining, we got the same results when compared with **Figures 4H, I**, all of which implies Exo_siMTA1 can enhance the tumor growth inhibition effect of GEM.

### HIF-α Is Responsible for EMT-Associated Chemoresistance in MTA1/EMT/Drug-Resistance Pathway

**Figure 6:**
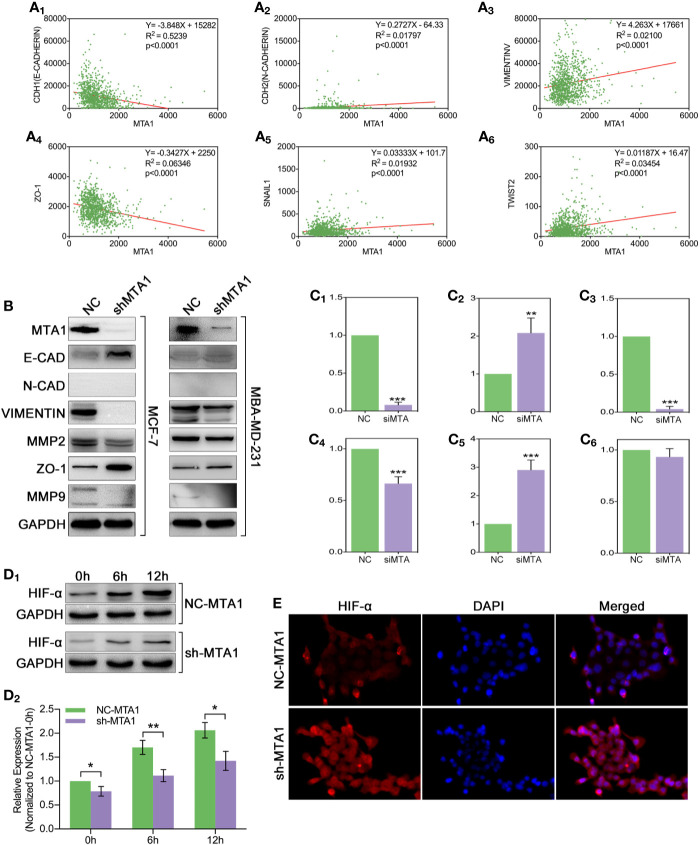
MTA1-knockdown down-regulates the EMT and EMT-associated HIF-α in MCF-7 cells. (1) Relationship between MTA1 and E-cadherin, N-cadherin, vimentin, ZO-1, snail1 and twist2, data from TCGA **(A_1-6_)**. (2) WB assay for EMT-associated protein in MCF-7 and MBA-MD-231 cell lines **(B, C_1-6_)**. (3) sh-MTA1 and NC-MTA1 cell lines of MCF-7 are treated with hypoxia for 0h, 6h and 12h, then detects the HIF-α level by WB **(D_1-2_)**. (4) Same treatment as in (3) which followed by IF assay **(E)**.

In order to explore the relationship between the MTA1 and EMT process, our study analyzed the correlation between the expression level of MTA1 and EMT-associated factors (EMT-AFs), such as E-cadherin, N-cadherin, vimentin, ZO-1, etc. As the TCGA data analysis show, there is positive correlation between MTA1 and “N-cadherin, vimentin, snail1 and twist2”, and negative correlation between MTA1 and “E-cadherin and ZO-1” (p<0.001, **Figures 6A_1-6_**). Then, through WB assay, we verified those results: MTA1 knockdown (p<0.001, **Figures 6B, C_1_**) led to up-regulation of E-cadherin (about 2-fold, p<0.001, **Figures 6B, C_2_**), and ZO-1 (about 3-fold, p<0.001, **Figures 6B, C_5_**), but down-regulation of vimentin (more than 90%, p<0.001, **Figures 6B, C_3_**), and MMP2 (about 70%, p<0.001, **Figures 6B, C_4_**), while here was few effects on the level of MMP9 (p>0.05, **Figures 6B, C_6_**) only in the MCF-7 cell line. Furthermore, we explored how MTA1 endows chemoresistance through EMT process. We detected the expression level of HIF-α which is involved in EMT-associated chemotherapeutic resistance. As the **Figures 6D_1-2_** show, MTA1 knockdown obviously down-regulated the expression of HIF-α by about 30% after 6h/12h-treatment of hypoxia in the MCF-7 cell line (6h: p<0.01; 12h: p<0.05). Subsequently, we achieved a similar result in the IF experiment (**Figures 6E**).

### Autophagy Is a Pivotal Role in MTA1-Induced Gemcitabine Resistance in MCF-7 Cells

**Figure 7:**
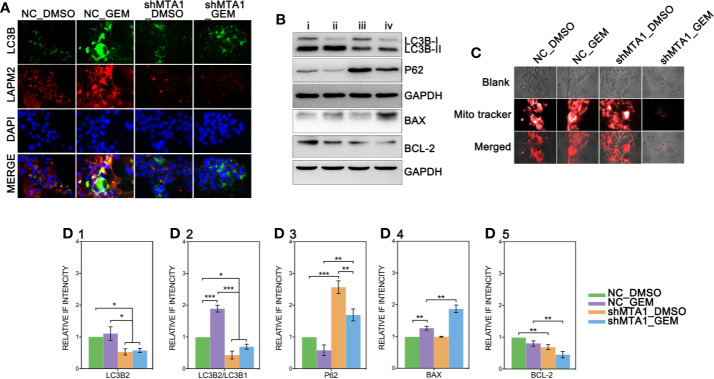
MTA1-knockdown restricts the autophagy process in MCF-7 cells. (1) NC_MCF-7 and shMTA1_MCF-7 cells are planted in 24-cell plates for 24h, followed by 48h-treatment of 5μM GEM or DMSO, then for IF assay for autophagy **(A)**. (2) NC_MCF-7 and shMTA1_MCF-7 cells are planted in 6-cell plates for 24h, followed by 48h-treatment of 5μM_GEM or DMSO (4 groups: NC_DMSO, NC_GEM, shMTA1_DMSO and shMTA1_GEM), then for WB assay for autophagy and apoptosis associated protein **(B, D_1-5_)**. (5) NC_MCF-7 and shMTA1_MCF-7 cells are planted in 24-cell plates for 24h, followed by 48h-treatment of 5μM_GEM or DMSO (4 groups: NC_DMSO, NC_GEM, shMTA1_DMSO and shMTA1_GEM), then for mitochondrial viability assay **(C)**.

Recent research shows that MTA1 can regulate the autophagy process, which leads to tamoxifen resistance in luminal-b breast cells. The down-regulated protein level of MTA1 leads to the down-regulation of autophagy, apoptosis and invasion in ovarian cancer cells. So, we tested the autophagy level after the MTA1 knockdown and the treatment of GEM. As the **Figure 7A** shows, LC3B was significantly up regulated in the NC_GEM group with very little difference in other 3 groups (NC_DMSO, shMTA1_DMSO and shMTA1_GEM). The expression level of LAMP2 was significantly up regulated in the NC_GEM group, and down regulated in the shMTA1_DMSO and shMTA1_GEM groups, as compared with the NC_DMSO group. In addition, WB assay showed about 50% down-regulation of LC3B-II expression in the shMTA1_DMSO and shMTA1_GEM groups when compared with the NC_DMSO and NC_GEM groups (p<0.05, **Figures 7B–D_1_**). Meanwhile, we found that MTA1 knockdown decreased the rate of LC3B-II/I with or without GEM (p<0.05, **Figure 7D_2_**), and increased the intracellular accumulation of p62 (p<0.001, **Figure 7D_3_**). We also found that MTA1 knockdown exactly restricted the GME-induced up-regulation of the LC3B-II/I and down-regulation of p62 (p<0.01, **Figures 7D_2-3_**). Furthermore, our study found that BAX is up-regulated about 1-fold and BCL-2 is down-regulated about 50% in the shMTA1_GEM group when compared with the NC_GEM group (p<0.01, **Figures 7D_4-5_**). BAX and BCL-2 are commonly known mitochondrial-associated proteins which are involved in apoptosis and autophagy pathways, so in the following experiments, our study tested the viability of mitochondria. As the **Figure 7C** shows, the mitochondria probe showed that combining treatment (MTA1 knockdown and GEM) significantly decreases the signal strength of live mitochondria.

## Discussion

The emerging of targeted drugs, such as pertuzumab and trastuzumab greatly prolong the overall survival in HER-2 positive breast cancer cases. However, for HER-2 negative type breast cancer, and especially for luminal-b type breast cancer and TNBC, the patients have seen few improvements to clinical treatment during the past decade. In addition, due to the excessive dependence on chemotherapy and endocrine therapy, those types of breast cancer are more prone to disease progression than HER 2-positive breast cancer and luminal-a type breast cancer. In advanced breast cancer, most patients are treated with first-line chemotherapeutics drugs and have to apply gemcitabine, capecitabine and other small molecule inhibitors to curb the further progression of breast cancer, so as to prolong the survival time with the disease as long as possible. Nevertheless, it is also easy for the rescue treatment of advanced breast cancer to quickly lead to drug resistance, thereby reducing the clinical efficacy. Therefore, further research on the mechanism of breast cancer chemotherapy resistance is still needed.

Although the mechanism of chemoresistance is complicated, some cellular processes have been considered as having important roles in chemoresistance. Cell cycle checkpoint is one of the above roles of tumors to reduce or repair the cellular toxicity induced by chemotherapeutic drugs (30, 31). In this process, tumor cells usually inhibit the activity of CDKs to make cells stay in the check-time to repair the cells (30). In the clinical treatment of advanced breast cancer, CDK4/6 inhibitors are applied to prevent disease progression. Autophagy, a famous member of chemotherapy resistance in breast cancer, pancreatic cancer, gastric cancer and other types of cancers, caused the tamoxifen resistance in luminal-b type breast cancer cell lines MCF-7 (14, 24, 32). HCQ and chloroquine (CQ), the autophagy inhibitors, have been used in clinical trials to treat advanced pancreatic cancer, myeloma and lung cancer, which have shown comparable effects in tumor restriction (8–11). The cell signaling pathways involved in autophagy are complicated. mTOR is the central regulator of autophagy, while the PI3K/Akt pathway, MAPK/Erk pathway and p53 pathway regulate the autophagy process by mTOR (5–7). Besides this, a recent study suggested that EMT, which is widely considered as having a key role in cell metastasis and invasion, is unnecessary for tumor invasion, but instead leads to gemcitabine tolerance in pancreatic cancer and cyclophosphamide resistance in breast cancer (16, 17). However, the EMT-mediated chemotherapy resistance is unclear. Previous studies show that there is the same protein in the regulation of the EMT process and CSC, which is involved in Wnt, Hedgehog and Notch pathways (18). The mesenchymal phenotype cells hold a more intensive trend of CSC than epithelial phenotypes. It is well known that CSC is important in chemotherapy resistance and tumor invasion, however some studies point out that EMT is irresponsible for the initiation of CSC due to the appearance of heterogenic CSC populations, containing mesenchymal-like CSCs (EMT CSCs) and epithelial-like CSCs (non-EMT CSCs) (33, 34). In addition, some studies point out that the up-regulation of EMT-associated transcriptional factors, containing Slug, Zeb1 and Twist1, can directly lead chemotherapy in breast cancer and NSCLC through the ATP-binding cassette (ABC) transporter family of proteins (ABCB1, ABCC1 and ABCG2) (35). Furthermore, the interaction of EMT and HIF-α has been reported in chemotherapy resistance (21). Tumor is just a camouflage of normal tissue, in which lots of proteins lose their original status, such as up-regulation, down-regulation and dislocation which endow abilities of chemoresistance. However, throughout the past few decades, information about gene-mediated chemotherapy resistance is still unclear, so further works are needed on the subject.

MTA1, a founding member of the metastasis-associated protein family, is first segregated from breast cancer cells, which is up-regulated in lots of types of tumors, such as breast cancer, ovarian cancer, colorectal cancer, pancreatic cancer and myeloma. This mediates the tumor regression, and chemotherapy tolerance, tumor invasion, radiation resistance and even endocrinotherapy failure are induced by MTA1 (23). In addition, according to the existing studies, MTA1 at least participates in the regulation of the Ras pathway, EMT process, Wnt pathway and MAPK/Erk pathway (23). A previous study shows that up-regulated expression of MTA1 leads tamoxifen resistance by promotion of autophagy in breast cancer (24). Besides, it has been evidenced that MTA1 participates in the regulation of HIF-α, by which MTA1 promotes tumor invasion and progression in pancreatic cancer and prostate cancer (36, 37). So, we guess that MTA1 is involved in autophagy-mediated and EMT-mediated chemotherapy resistance. In our study, we found that the mRNA level of MTA1 is higher in breast cancer, as compared with normal tissue through TCGA debase analysis, and the clinical data showed that MTA1 was up-regulated in samples among the clinical stage-III/IV, when compared with samples in stage-I/II (**Figure 1M**). In addition, overall survival analysis showed that MTA1 was a positive factor for breast cancer regression (p=0.0289, **Figure 1N**). In the following experiments, we found that down-regulation of MTA1 enhances the tumor growth inhibition effect from GEM in MCF-7 cells *in vitro* (**Figure 2A_4_**) through enhancing cell apoptosis and ros-reaction levels (**Figure 3**). Meanwhile, MTA1 knockdown even maintained the gemcitabine-mediated growth inhibition after being withdrawn *in vitro* and vivo (**Figure 2H_1_** and **Figure 4D**). Furthermore, in order to make possible the application of the MTA1 targeted treatment in luminal-b type breast cancer in clinic, our study generated siMTA1-loaded exosomes. Interestingly, our data show that, MTA1-loaded exosomes successfully enhanced the GEM-mediated tumor growth inhibition effect (**Figure 5**). Then, our study explored the mechanism of MTA1-induced chemotherapy tolerance. Our study found that MTA1 knockdown exactly inhibited the GEM-induced autophagy and enhanced the mitochondrial damage. Besides, MTA1 knockdown reverted the EMT process, in which we found up-regulated levels of E-cadherin and ZO-1 and down-regulated levels of vimentin and MMP2 (**Figures 6B, C_1-5_**). Furthermore, we found that the MTA1-knockdown-induced mesenchymal phenotype held a lower level of HIF-α after hypoxia treatment. So, we infer that MTA1 mediated chemotherapy resistance through EMT and the autophagy pathway.

Overall, our study reveals that: (1): Higher level of MTA1 accompanies worse prognosis in luminal-b type of breast cancer by analysis of TCGA debase and clinical samples; (2) MTA1 knockdown sensitizes the short-term and withdrawal chemotherapeutic effects of GEM in the MCF-7 cell line *in vitro* and *in vivo*. (3) siMTA1-loaded exosomes enhance the tumor growth inhibition effect of GEM *in vivo*. (4) MTA1 knockdown reverts the EMT/HIF-α process and restricts the autophagy process, which are important in MTA1-mediated chemotherapy. However, we do not explore the regulation process between cancer stem cells and EMT by MTA1, and this will be explored in our following works.

## Data Availability Statement

The raw data supporting the conclusions of this article will be made available by the authors, without undue reservation, to any qualified researcher.

## Ethics Statement

The studies involving human participants were reviewed and approved by The First Affiliated Hospital of Anhui Medical University Review Board and the ethics committees of Anhui Medical University. Written informed consent for participation was not required for this study in accordance with the national legislation and the institutional requirements. The animal study was reviewed and approved by The First Affiliated Hospital of Anhui Medical University Review Board and the ethics committees of Anhui Medical University.

## Author Contributions

All authors listed have made a substantial, direct, and intellectual contribution to the work and approved it for publication.

## Funding

This study is supported by Key Research and Development Plan Projects of Anhui Province (Project Nos.201904a07020045).

## Conflict of Interest

The authors declare that the research was conducted in the absence of any commercial or financial relationships that could be construed as a potential conflict of interest.
